# Determining distinct roles of IL-1α through generation of an IL-1α knockout mouse with no defect in IL-1β expression

**DOI:** 10.3389/fimmu.2022.1068230

**Published:** 2022-11-24

**Authors:** R.K. Subbarao Malireddi, Ratnakar R. Bynigeri, Balabhaskararao Kancharana, Bhesh Raj Sharma, Amanda R. Burton, Stephane Pelletier, Thirumala-Devi Kanneganti

**Affiliations:** Department of Immunology, St. Jude Children’s Research Hospital, Memphis, TN, United States

**Keywords:** PAMP, innate immunity, inflammation, inflammasome, IL-1α, IL-1β, CXCL1, caspase-1

## Abstract

Interleukin 1α (IL-1α) and IL-1β are the founding members of the IL-1 cytokine family, and these innate immune inflammatory mediators are critically important in health and disease. Early studies on these molecules suggested that their expression was interdependent, with an initial genetic model of IL-1α depletion, the IL-1α KO mouse (*Il1a*-KO^line1^), showing reduced IL-1β expression. However, studies using this line in models of infection and inflammation resulted in contrasting observations. To overcome the limitations of this genetic model, we have generated and characterized a new line of IL-1α KO mice (*Il1a*-KO^line2^) using CRISPR-Cas9 technology. In contrast to cells from *Il1a*-KO^line1^, where IL-1β expression was drastically reduced, bone marrow-derived macrophages (BMDMs) from *Il1a*-KO^line2^ mice showed normal induction and activation of IL-1β. Additionally, *Il1a*-KO^line2^ BMDMs showed normal inflammasome activation and IL-1β expression in response to multiple innate immune triggers, including both pathogen-associated molecular patterns and pathogens. Moreover, using *Il1a*-KO^line2^ cells, we confirmed that IL-1α, independent of IL-1β, is critical for the expression of the neutrophil chemoattractant KC/CXCL1. Overall, we report the generation of a new line of IL-1α KO mice and confirm functions for IL-1α independent of IL-1β. Future studies on the unique functions of IL-1α and IL-1β using these mice will be critical to identify new roles for these molecules in health and disease and develop therapeutic strategies.

## Introduction

The IL-1 family of cytokines is a diverse family made up of potent inducers of inflammation. Members of this family can either prevent or promote disease, and they have been widely recognized as potential therapeutic targets (1–5). The three members of the IL-1 sub-family, IL-1α, IL-1β, and IL-1 receptor antagonist (IL-1RA), bind the same IL-1 receptor (IL-1R). The cytokines IL-1α and IL-1β act as agonistic ligands, whereas IL-1RA is a strong antagonist; together, these molecules orchestrate robust proinflammatory immune responses (6, 7).

Among the IL-1 cytokines, significant overlap has been observed in the downstream processes they activate. However, there are also key differences between their expression and release and the biological processes they drive (8). The pro-form of IL-1β is biologically inactive and requires proteolytic processing for its activation. Inflammasome-dependent caspase-1 activation and pyroptosis are the major mechanisms responsible for IL-1β processing and release (9–11). Unlike IL-1β, the pro-form of IL-1α is constitutively expressed in most cells from healthy hosts (12, 13); it is also biologically active and can be present directly on the plasma membrane for signaling or released following membrane damage during various forms of cell death, making it a classic danger signal (5, 14, 15).

As signaling molecules, a wide range of pathogen-associated and damage-associated molecular patterns (PAMPs and DAMPs) that activate innate immune signaling induce the expression and activation of both IL-1α and IL-1β (5, 16). IL-1 family receptors carry the cytoplasmic TIR domain, a shared feature with pathogen sensing toll-like receptors (TLRs), making them excellent amplifiers of inflammatory signaling (17). Indeed, nanomolar doses of IL-1α and IL-1β can trigger lethal inflammatory responses in mice and humans (18–20). Consistently, IL-1α and IL-1β were shown to act as self-amplifying factors and upregulate each other *via* IL-1R signaling (7, 21–24). However, studies of IL-1α and IL-1β have produced conflicting results with regard to how these cytokines regulate each other. Studies focused on TLR triggers reported that these self-amplifying positive feedback mechanisms are redundant or not important to amplify the production of IL-1α and IL-1β further (25–29). These observations differed from studies using a genetic *Il1a* knockout mice (hereafter referred to as *Il1a*-KO^line1^), which showed substantial reduction in IL-1β production when *Il1a* was deleted (30–32), suggesting that IL-1α may regulate IL-1β expression even during TLR activation. These conclusions remained debated and poorly understood for many years.

Therefore, we sought to generate a new line of *Il1a* knockout mice (hereafter referred to as *Il1a*-KO^line2^) using CRISPR-Cas9 technology. The newly generated *Il1a*-KO^line2^ mice showed normal development, with comparable levels of basal immune cells in the blood compared with wild-type (WT) mice. Bone marrow-derived macrophages (BMDMs) prepared from the *Il1a*-KO^line2^ mice showed no defect in expression or activation of inflammasome components in response to PAMPs and live pathogen triggers. Additionally, while the cells from *Il1a*-KO^line1^ showed reduced expression of both IL-1α and IL-1β, *Il1a*-KO^line2^ macrophages had no expression of IL-1α but near-normal expression of IL-1β. Moreover, the *Il1a*-KO^line2^ BMDMs showed a specific requirement of IL-1α for the expression of neutrophil chemoattractant KC/CXCL1, further confirming the functional accuracy of the KO. In summary, we generated and characterized a new line of IL-1α KO mice that improves upon the previous version and has normal IL-1β expression. These mice can be broadly used for future studies on the unique functions of IL-1α and IL-1β to establish their relevance in health and disease and identify new treatment strategies.

## Results

### Generation of the IL-1α KO (*Il1a*-KO^line2^) mouse using CRISPR/Cas9 technology

**Figure 1:**
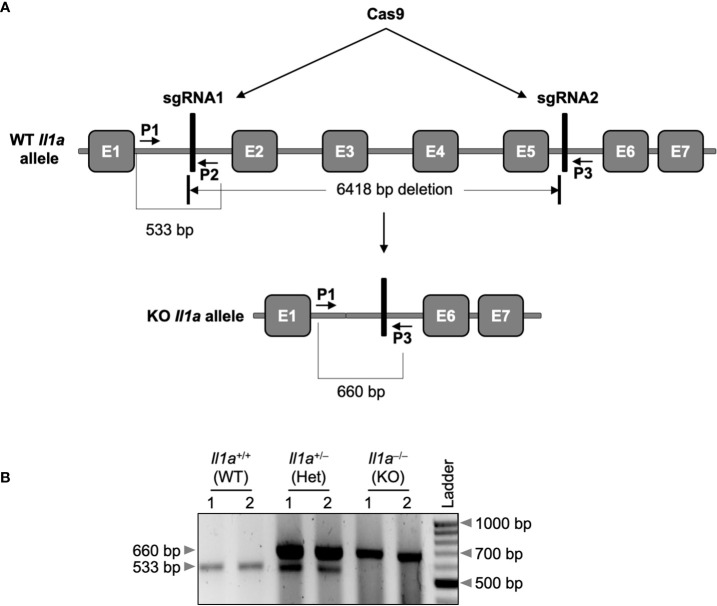
Generation of the *Il1a* ^–/–^ (*Il1a*-KO^line2^) mouse using CRISPR/Cas9 technology. **(A)** Two sgRNAs targeting the *Il1a* locus were designed and used to delete exons 2 to 5 (E2 to E5), as described in the materials and methods section. The vertical bars denote the sgRNAs 1 and 2, respectively (depictions are not to scale) in the genomic sequence. The location of the deleted genomic fragment and the primer-binding locations are depicted using short arrows. **(B)** The PCR amplification of the *Il1a* locus from the DNA of wild-type (WT), heterozygous, (Het), or knockout (KO) mice using the primers (primers P1 and P2 together with P3).

**Figure 2:**
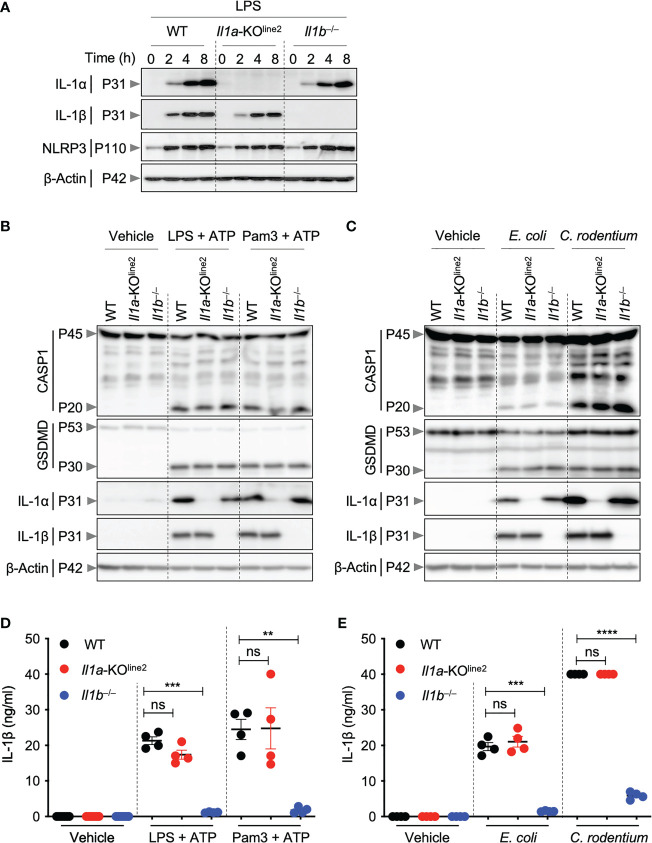
CRISPR-based genetic deletion of *Il1a* (*Il1a*-KO^line2^) does not affect IL-1β expression or activation. **(A)** Western blot analysis of pro–IL-1α (P31), pro–IL-1β (P31), NLRP3 (P110), and β-Actin (P42) in bone marrow-derived macrophages (BMDMs) treated with lipopolysaccharide (LPS) for indicated times. **(B, C)** Western blot analysis of pro- (P45) and activated (P20) caspase-1 (CASP1), pro- (P53) and activated (P30) gasdermin D (GSDMD), pro–IL-1α (P31), pro–IL-1β (P31), and β-Actin (P42) in BMDMs treated with LPS + ATP or Pam3CSK4 (Pam3) + ATP for 4 h **(B)**, or BMDMs infected with *E*. *coli* or *C*. *rodentium* for 24 h **(C)**. **(D, E)** Measurement of IL-1β release in the cellular supernatants collected from BMDMs treated as detailed in panels **(B)** and **(C)**, respectively, for **(D)** and **(E)**. Western blot of β-actin was used as loading control. Data are representative of at least two independent experiments **(A–E)**. Data are presented as the mean ± SEM **(D, E)**. Analyses of the *P* values were performed using the *t* test **(D, E)**. ns, non-significant; **P < 0.01; ***P < 0.001; ****P < 0.0001.

Although IL-1α has long been recognized as a critical regulator of inflammation and immune responses (8), its specific functions in physiologic and pathologic inflammatory outcomes in health and disease remain unclear. IL-1α is subjected to complex regulation, and early genetic studies using different knockout mice produced conflicting observations (27, 30–32). To clarify the previously observed contradictory roles of IL-1α in IL-1β expression in *Il1a*-KO^line1^ mice, we generated a new line of IL-1α knockout (KO) mice using CRISPR-Cas9 technology, referred to here as *Il1a*-KO^line2^ (**Figure 1**). Exons 2-5 of the *Il1a* gene were deleted by using simultaneous injection of two individual gRNAs with human codon optimized Cas9 mRNA (**Figure 1A**). We opted to use pronuclear-staged C57BL/6J zygotes for the injections to minimize the background-related genetic issues. Successful generation of the *Il1a*-deficient mice was assessed by targeted deep sequencing and further confirmed by PCR amplification of genomic DNA from the WT and mutant alleles (**Figure 1B**), and western blot analysis to confirm the loss of IL-1α protein production (**Figure 2A**). Additionally, because IL-1α is a multifaceted cytokine that we postulated may have a role in regulating immune cell phenotypes at basal levels, we evaluated the immune cellularity in the blood from the newly generated CRISPR *Il1a* ^–/–^ mice (*Il1a*-KO^line2^). We found that these mice did not show any gross abnormalities in the immune cellularity (**Supplementary Figures 1A, B**). In sum, we generated a new line of IL-1α knockout mice, *Il1a*-KO^line2^, and confirmed the loss of IL-1α expression with no defects in overall blood immune cellularity.

### CRISPR-based genetic deletion of *Il1a* does not affect IL-1β expression or activation

Both IL-1α and IL-1β are known to be highly induced in response to pathogenic insults. Therefore, we next sought to characterize the cytokine expression in cells from the newly generated *Il1a*-KO^line2^ mice in response to PAMPs and pathogens. Treatment of BMDMs with lipopolysaccharide (LPS, a toll-like receptor 4 (TLR4) agonist from Gram-negative bacteria) induced robust and time-dependent expression of IL-1α protein in WT cells but not in *Il1a*-KO^line2^ cells (**Figure 2A**). In addition, the induction of IL-1α protein expression was not affected by *Il1b* genetic deletion, and the expression of IL-1β in response to LPS was similar in the WT and *Il1a*-KO^line2^ cells (**Figure 2A**). In contrast, we observed a delay and reduction in the production of IL-1β in the previously generated *Il1a*-KO^line1^ cells in response to LPS (**Supplementary Figure 2A**).

We next sought to further understand the potential roles for IL-1α and IL-1β in NLRP3 inflammasome priming, which is known to produce mature IL-1β. We found that IL-1α was not required for upregulation of NLRP3 or IL-1β expression in response to the innate immune triggers LPS, LPS plus ATP, Pam3CSK4 (Pam3) plus ATP, or Gram-negative bacteria *Escherichia coli* or *Citrobacter rodentium* (**Figures 2A–C**). Moreover, the activation of canonical and non-canonical inflammasomes, as measured by cleavage of caspase-1 and gasdermin D (GSDMD), was not reduced by deletion of *Il1a* (**Figures 2B, C**). Consistently, IL-1β release was similar in WT and *Il1a*-KO^line2^ BMDMs (**Figures 2D, E**). In contrast, using similar experimental approaches, we observed defects in IL-1β expression in macrophages from the earlier *Il1a*-KO^line1^ line, with pronounced reductions in IL-1β expression at early time points in response to LPS, while the induction improved at later timepoints (**Supplementary Figure 2A**). We also observed reductions in IL-1β expression in response to NLRP3 inflammasome triggers LPS plus ATP and Pam3 plus ATP (**Supplementary Figure 2B**). We did not observe defects in NLRP3 production or caspase-1 and GSDMD activation in *Il1a*-KO^line1^ cells (**Supplementary Figures 2A, B**). Together, these results show that while the previously generated *Il1a*-KO^line1^ mice had defects in IL-1β production, *Il1a*-KO^line2^ mice did not share these defects.

### CRISPR-based genetic deletion of *Il1a* confirms its critical role in the expression of the chemokine KC (CXCL1)

**Figure 3:**
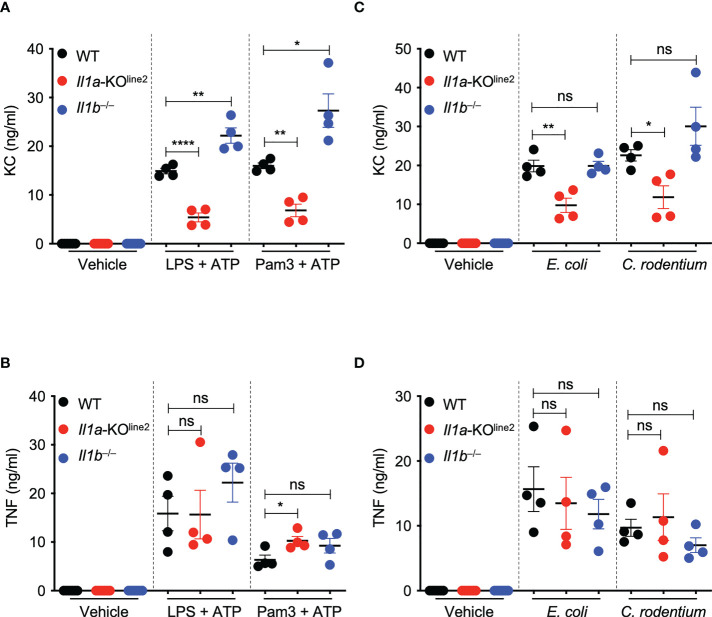
CRISPR-based genetic deletion of *Il1a* (*Il1a*-KO^line2^) confirms its critical role in the expression of the chemokine KC (CXCL1). **(A–D)** Measurement of secreted cytokines KC and TNF in bone marrow-derived macrophages (BMDMs) treated with lipopolysaccharide (LPS) + ATP or Pam3CSK4 (Pam3) + ATP for 4 h **(A, B)** or infected with *E. coli* or *C. rodentium* for 24 h **(C, D)**. Data are representative of at least two independent experiments **(A–D)**. Data are presented as the mean ± SEM **(A–D)**. Analyses of the *P* values were performed using the *t* test **(A–D)**. ns, non-significant; *P < 0.05; **P < 0.01; ****P < 0.0001.

IL-1α is a pleiotropic cytokine and critical amplifier of inflammation in response to both infection and sterile cellular insults (8). IL-1α also plays key roles in regulating neutrophil-chemotactic factors such as the chemokine KC (CXCL1) in mice (33). Therefore, to further confirm the IL-1α deletion in the newly generated *Il1a*-KO^line2^ mice and assess its functional effects, we evaluated expression of TNF and KC in response to innate immune triggers. We specifically used LPS plus ATP as the stimulation to mimic the inflammasome activation conditions used above to determine differences in IL-1β production, as this stimulus has been previously shown to be suitable for measuring inflammatory markers (34). We found that IL-1α specifically was required to produce KC, but not TNF, in response to both PAMP- and pathogen-induced signaling in macrophages; loss of IL-1α resulted in significant decreases in KC release, while loss of IL-1β did not decrease KC release (**Figures 3A–D**). Instead, we observed significantly increased levels of KC production in *Il1b* ^−⁄−^ cells in response to LPS plus ATP and Pam3 plus ATP treatments (**Figure 3A**), suggesting a competition between IL-1α and IL-1β for IL-1R binding in this context, where the increased availability of IL-1R molecules for binding by IL-1α may promote hyper-expression of select inflammatory factors in the absence of IL-1β. Together, these findings confirm the specific role of IL-1α for the release of KC, further supporting the functional relevance of the newly created *Il1a*-KO^line2^ mice for the evaluation of IL-1α–mediated signaling and disease phenotypes.

## Discussion

Members of the IL-1 family of cytokines play important roles as inflammatory mediators in host defense but have also been implicated in disease pathogenesis. Therefore, understanding the distinct functions of IL-1 family members is fundamental to our understanding of the molecular basis of disease. Previous genetic models of IL-1α deletion have displayed defects in IL-1β production, making it difficult to determine the distinct roles of these molecules in immune responses. To overcome this obstacle, we report the generation of a genetic deletion of *Il1a* in mice using CRISPR technology that did not affect IL-1β induction in response to microbial PAMPs and pathogens. Our findings suggest that expression of IL-1β in response to TLR activation is not affected by loss of IL-1α.

Growing evidence supports that IL-1α and IL-1β have distinct functions (35–37). Our findings further confirm that IL-1α is a non-redundant positive regulator of the expression of the chemokine KC in macrophages, which is consistent with earlier studies reporting the specific role of IL-1α in promoting production and recruitment of neutrophils in chronic inflammatory conditions (33, 38–40). The mechanism behind the differences between IL-1α and IL-1β in regulating KC requires further study but may be driven by the distinct localization of these cytokines. IL-1α is unique in its ability to localize to the nucleus and directly regulate transcription (7), and it may selectively modulate the transcription of the *Cxcl1* gene. However, IL-1β has also been shown to be important for the induction of neutrophil growth- and chemotactic-factors (41–45). Therefore, it is plausible that IL-1β might also contribute to neutrophil-mediated inflammatory conditions as a result of the activation of cell death modalities that drive IL-1β maturation *via* the activation of caspase-1 or other proteases (46), though this requires further study.

Additionally, previous studies using the earlier *Il1a*-KO^line1^ mice distinguished unique functions of IL-1α and IL-1β in the development of chronic autoinflammatory diseases (40, 42, 43, 47, 48). Our results suggest that the ability to use the *Il1a*-KO^line1^ mice to successfully identify this differential phenotype is due to the chronic nature of the disease. We found that the reduction of IL-1β expression in *Il1a*-KO^line1^ cells was pronounced only at early time points following stimulation, and that prolonged stimulation resulted in similar levels of IL-1β in WT and *Il1a*-KO^line1^ cells, in response to both PAMPs and pathogens. This suggests that WT and *Il1a*-KO^line1^ mice would have similar levels of IL-1β during chronic disease, allowing differential phenotypes between *Il1b* ^−⁄−^ and *Il1a* ^−⁄−^ mice to be observed.

Given the critical roles of IL-1 family cytokines in inflammation and pathology, these cytokines have been targeted in several therapeutic strategies which have further highlighted unique functions for IL-1α and IL-1β. For example, the recent SAVE-MORE trial showed that anakinra, which blocks both IL-1α and IL-1β, reduced the risk of clinical progression in patients with COVID-19, when co-administered with dexamethasone (49). Accordingly, anakinra was authorized for the treatment of COVID-19 in Europe by the EMA. In contrast, the CAN-COVID trial, which was designed to evaluate the efficacy of canakinumab (a specific IL-1β blocking antibody) failed to improve the survival of patients with COVID-19 (50). These studies further expand the concept that IL-1α plays a dominant and potentially specific role in driving IL-1β-independent inflammatory immune responses and pathology in some contexts.

Together, these observations show that caution should be used when interpreting previous studies and highlight the need to authenticate genetic resources for future work. The development of the *Il1a*-KO^line2^ mouse line, which does not seem to display acute or chronic defects in IL-1β production, may help address many of the critical, long-standing questions in the field of immunobiology regarding the shared and unique functions and context-dependent interdependencies of IL-1α and IL-1β cytokines to improve understanding of the molecular basis of disease and inform therapeutic strategies.

## Materials and methods

### Mice

*Il1b* ^−⁄−^ (51) and *Il1a* ^−⁄−^ (*Il1a*-KO^line1^) (52) mice were both previously described. *Il1a* ^−⁄−^ (*Il1a*-KO^line2^) mice were generated in the current study and are described below. All mice were generated on or extensively backcrossed to the C57BL/6 background. All mice were bred at the Animal Resources Center at St. Jude Children’s Research Hospital and maintained under specific pathogen-free conditions. Mice were maintained with a 12 h light/dark cycle and were fed standard chow. Animal studies were conducted under protocols approved by the St. Jude Children’s Research Hospital committee on the Use and Care of Animals.

### Generation of the new IL-1α KO *(Il1a*-KO^line2^) mouse strain

The new *Il1a*-KO^line2^ mouse was generated using CRISPR/Cas9 technology in collaboration with the St. Jude Transgenic/Gene Knockout Shared Resource facility. Pronuclear-staged C57BL/6J zygotes were injected with human codon-optimized *Cas9* mRNA transcripts (50 ng/μl) combined with two guide RNAs (120 ng/μl each; sgRNA1 for the 5’ of exon 2: AAAAGCTTCTGACGTACCACagg, and sgRNA2 for the 3’ of exon 5: AAGTAACAGCGGAGCGCTTTtgg (pam sequences are underlined)) to generate a long deletion encompassing exons (E) 2–5 of the *Il1a* gene (**Figure 1A**). Zygotes were surgically transplanted into the oviducts of pseudo-pregnant CD1 females, and newborn mice carrying the desired deletion in the *Il1a* allele were identified by PCR agarose gel-electrophoresis (**Figure 1B**) and Sanger sequencing. The WT allele was PCR amplified by using the primers IL1a_F1 (5’-GGGCACACGAATTCACACTCACA-3’; primer P1) and IL1a_R1 (5’-GGAGAACTTGGTTCCTGTTAGGGTGA-3’; primer P2), and the KO allele was amplified by using IL1a_F1 and IL1a_R2 (5’- TGATTAGCTTCCTTTGGGCTTTGA-3’; primer P3) primer pairs. The details of the generation of the CRISPR reagents were described previously (53). The uniqueness of sgRNAs and the off-target sites with fewer than three mismatches were found using the Cas-OFFinder algorithm (54).

### Macrophage differentiation and stimulation

BMDMs were prepared as described previously (55). In short, bone marrow cells were cultured in IMDM supplemented with 30% L929 cell-conditioned medium, 10% FBS, 1% nonessential amino acids, and 1% penicillin-streptomycin for 6 days to differentiate into macrophages. On day 6, BMDMs were counted and seeded at 10^6^ cells per well in 12-well culture plates in DMEM containing 10% FBS, 1% nonessential amino acids, and 1% penicillin-streptomycin. iBMDMs (immortalized BMDMs from *Il1a* ^−⁄−^ (*Il1a*-KO^line1^) mice) were maintained in DMEM supplemented with 5% L929 cell-conditioned medium, 10% FBS, 1% nonessential amino acid, and 1% penicillin-streptomycin. Stimulations were performed with LPS alone (100 ng/ml) for the indicated times, LPS (100 ng/ml) or Pam3 (1 µg/ml) for 3.5 h followed by the addition of ATP (5 mM final concentration) for 30 min, or *E. coli* (MOI, 20) or *C. rodentium* (MOI, 20) for 24 h.

### Flow cytometry and analysis of cellularity

The cellular phenotypes of immune cells in the blood were analyzed either by flow cytometry (for T cell subsets and B cells) or by using an automated hematology analyzer machine (for % lymphocytes, % neutrophils, % monocytes, red blood cell (RBC) counts, hemoglobin (HB) quantification, and platelet (PLT) quantification). The following antibodies were used for cell staining: anti-CD19 (APC, clone ID3), anti-CD45.2 (FITC, clone 104), and anti-TCRβ (PECy7, clone H57-597) from Biolegend, and anti-CD8a (efluor450, clone 53-6.7) from eBiosciences. Samples were assessed and data were acquired on LSR II Flow Cytometer from BD Biosciences and analyzed using the FlowJo software (Tree Star), version 10.2 (FlowJo LLC).

### Western blotting

Samples for immunoblotting of caspase-1 were prepared by mixing the cell lysates with culture supernatants (lysis buffer: 5% NP-40 solution in water supplemented with 10 mM DTT and protease inhibitor solution at 1× final concentration); samples for all other protein immunoblotting were prepared without the supernatants in RIPA lysis buffer. Samples were mixed and denatured in loading buffer containing SDS and 100 mM DTT and boiled for 12 min. SDS-PAGE–separated proteins were transferred to PVDF membranes and immunoblotted with primary antibodies against IL-1α (503207, Biolegend), IL-1β (12426, Cell Signaling Technology), caspase-1 (AG-20B-0042, Adipogen), NLRP3 (AG-20B-0014, Adipogen), GSDMD (ab209845, Abcam), and β-Actin (sc-47778 HRP, Santa Cruz), Appropriate horseradish peroxidase (HRP)–conjugated secondary antibodies (anti-Armenian hamster [127-035-099], anti-mouse [315-035-047], and anti-rabbit [111-035-047], Jackson ImmunoResearch Laboratories) were used as described previously (56). Immunoblot images were acquired on an Amersham Imager using Immobilon^®^ Forte Western HRP Substrate (WBLUF0500, Millipore).

### Cytokine analysis

Cytokines and chemokines were measured by multiplex ELISA (Millipore), as per the manufacturer's instructions.

### Statistical analysis

GraphPad Prism 9.0 software was used for data analysis. Data are presented as mean ± SEM. Statistical significance was determined by *t* tests (two-tailed) for two groups.

## Data availability statement

The original contributions presented in the study are included in the article/**Supplementary Material**. Further inquiries can be directed to the corresponding author.

## Ethics statement

The animal study was reviewed and approved by the St. Jude Children’s Research Hospital committee on the Use and Care of Animals.

## Author contributions

RKSM and T-DK designed the study. RKSM, RRB, BK, and BS performed experiments. AB and SP performed the CRISPR-based knockout generation and initial breeding. RKSM, RRB, and T-DK analyzed the data. RKSM and RRB wrote the manuscript with input from all authors. T-DK oversaw the project. All authors contributed to the article and approved the submitted version.

## Funding

Work from our laboratory is supported by the US National Institutes of Health (AI101935, AI124346, AI160179, AR056296, and CA253095 to T-DK) and the American Lebanese Syrian Associated Charities (to T-DK). The content is solely the responsibility of the authors and does not necessarily represent the official views of the National Institutes of Health.

## Acknowledgments

We thank all the members of the Kanneganti laboratory for their comments and suggestions during the development of this manuscript. We thank R. Tweedell, PhD, and J. Gullett, PhD, for scientific editing and writing support, and Katie Combs and Lauren Kneeland for mouse colony support.

## Conflict of interest

T-DK is a consultant for Pfizer.

The remaining authors declare that the research was conducted in the absence of any commercial or financial relationships that could be construed as a potential conflict of interest.

## Publisher’s note

All claims expressed in this article are solely those of the authors and do not necessarily represent those of their affiliated organizations, or those of the publisher, the editors and the reviewers. Any product that may be evaluated in this article, or claim that may be made by its manufacturer, is not guaranteed or endorsed by the publisher.

## References

[1] Ridker PM Thuren T Zalewski A Libby P. Interleukin-1beta inhibition and the prevention of recurrent cardiovascular events: rationale and design of the canakinumab anti-inflammatory thrombosis outcomes study (CANTOS). Am Heart J (2011) 162:597–605. doi: 10.1016/j.ahj.2011.06.012 21982649

[2] Lukens JR Gross JM Kanneganti TD. IL-1 family cytokines trigger sterile inflammatory disease. Front Immunol (2012) 3:315. doi: 10.3389/fimmu.2012.00315 23087690 PMC3466588

[3] Ridker PM Everett BM Thuren T Macfadyen JG Chang WH Ballantyne C. Antiinflammatory therapy with canakinumab for atherosclerotic disease. N Engl J Med (2017) 377:1119–31. doi: 10.1056/NEJMoa1707914 28845751

[4] Ridker PM Macfadyen JG Thuren T Everett BM Libby P Glynn RJ. Effect of interleukin-1beta inhibition with canakinumab on incident lung cancer in patients with atherosclerosis: exploratory results from a randomised, double-blind, placebo-controlled trial. Lancet (2017) 390:1833–42. doi: 10.1016/S0140-6736(17)32247-X 28855077

[5] Malik A Kanneganti TD. Function and regulation of IL-1alpha in inflammatory diseases and cancer. Immunol Rev (2018) 281:124–37. doi: 10.1111/imr.12615 PMC5739076 29247991

[6] Dinarello CA Goldin NP Wolff SM. Demonstration and characterization of two distinct human leukocytic pyrogens. J Exp Med (1974) 139:1369–81. doi: 10.1084/jem.139.6.1369 PMC2139679 4829934

[7] Dinarello CA. Immunological and inflammatory functions of the interleukin-1 family. Annu Rev Immunol (2009) 27:519–50. doi: 10.1146/annurev.immunol.021908.132612 19302047

[8] Cavalli G Colafrancesco S Emmi G Imazio M Lopalco G Maggio MC. Interleukin 1alpha: a comprehensive review on the role of IL-1alpha in the pathogenesis and treatment of autoimmune and inflammatory diseases. Autoimmun Rev (2021) 20:102763. doi: 10.1016/j.autrev.2021.102763 33482337

[9] Kanneganti TD. Central roles of NLRs and inflammasomes in viral infection. Nat Rev Immunol (2010) 10:688–98. doi: 10.1038/nri2851 PMC3909537 20847744

[10] Kayagaki N Stowe IB Lee BL O’rourke K Anderson K Warming S. Caspase-11 cleaves gasdermin d for non-canonical inflammasome signalling. Nature (2015) 526:666–71. doi: 10.1038/nature15541 26375259

[11] Shi J Zhao Y Wang K Shi X Wang Y Huang H. Cleavage of GSDMD by inflammatory caspases determines pyroptotic cell death. Nature (2015) 526:660–5. doi: 10.1038/nature15514 26375003

[12] Kupper TS Ballard DW Chua AO Mcguire JS Flood PM Horowitz MC. Human keratinocytes contain mRNA indistinguishable from monocyte interleukin 1 alpha and beta mRNA. Keratinocyte epidermal cell-derived thymocyte-activating factor is identical to interleukin 1. J Exp Med (1986) 164:2095–100. doi: 10.1084/jem.164.6.2095 PMC2188493 2431094

[13] Berda-Haddad Y Robert S Salers P Zekraoui L Farnarier C Dinarello CA. Sterile inflammation of endothelial cell-derived apoptotic bodies is mediated by interleukin-1alpha. Proc Natl Acad Sci U.S.A. (2011) 108:20684–9. doi: 10.1073/pnas.1116848108 PMC3251090 22143786

[14] Kurt-Jones EA Beller DI Mizel SB Unanue ER. Identification of a membrane-associated interleukin 1 in macrophages. Proc Natl Acad Sci U.S.A. (1985) 82:1204–8. doi: 10.1073/pnas.82.4.1204 PMC397223 3919388

[15] Kaplanski G Farnarier C Kaplanski S Porat R Shapiro L Bongrand P. Interleukin-1 induces interleukin-8 secretion from endothelial cells by a juxtacrine mechanism. Blood (1994) 84:4242–8. doi: 10.1182/blood.V84.12.4242.bloodjournal84124242 7994038

[16] Mantovani A Dinarello CA Molgora M Garlanda C. Interleukin-1 and related cytokines in the regulation of inflammation and immunity. Immunity (2019) 50:778–95. doi: 10.1016/j.immuni.2019.03.012 PMC7174020 30995499

[17] Boraschi D Italiani P Weil S Martin MU. The family of the interleukin-1 receptors. Immunol Rev (2018) 281:197–232. doi: 10.1111/imr.12606 29248002

[18] Lomedico PT Gubler U Hellmann CP Dukovich M Giri JG Pan YC. Cloning and expression of murine interleukin-1 cDNA in Escherichia coli. Nature (1984) 312:458–62. doi: 10.1038/312458a0 6209582

[19] Smith JW Longo DL Alvord WG Janik JE Sharfman WH Gause BL. The effects of treatment with interleukin-1 alpha on platelet recovery after high-dose carboplatin. N Engl J Med (1993) 328:756–61. doi: 10.1056/NEJM199303183281103 8437596

[20] Dinarello CA. Biologic basis for interleukin-1 in disease. Blood (1996) 87:2095–147. doi: 10.1182/blood.V87.6.2095.bloodjournal8762095 8630372

[21] Dinarello CA Ikejima T Warner SJ Orencole SF Lonnemann G Cannon JG. Interleukin 1 induces interleukin 1. I. Induction of circulating interleukin 1 in rabbits *in vivo* and in human mononuclear cells *in vitro* . J Immunol (1987) 139:1902–10. 3497982

[22] Warner SJ Auger KR Libby P. Interleukin 1 induces interleukin 1. II. Recombinant human interleukin 1 induces interleukin 1 production by adult human vascular endothelial cells. J Immunol (1987) 139:1911–7. 3497983

[23] Goldbach-Mansky R Dailey NJ Canna SW Gelabert A Jones J Rubin BI. Neonatal-onset multisystem inflammatory disease responsive to interleukin-1beta inhibition. N Engl J Med (2006) 355:581–92. doi: 10.1056/NEJMoa055137 PMC4178954 16899778

[24] Greten FR Arkan MC Bollrath J Hsu LC Goode J Miething C. NF-kappaB is a negative regulator of IL-1beta secretion as revealed by genetic and pharmacological inhibition of IKKbeta. Cell (2007) 130:918–31. doi: 10.1016/j.cell.2007.07.009 PMC2134986 17803913

[25] Glaccum MB Stocking KL Charrier K Smith JL Willis CR Maliszewski C. Phenotypic and functional characterization of mice that lack the type I receptor for IL-1. J Immunol (1997) 159:3364–71. 9317135

[26] Labow M Shuster D Zetterstrom M Nunes P Terry R Cullinan EB. Absence of IL-1 signaling and reduced inflammatory response in IL-1 type I receptor-deficient mice. J Immunol (1997) 159:2452–61. 9278338

[27] Fettelschoss A Kistowska M Leibundgut-Landmann S Beer HD Johansen P Senti G. Inflammasome activation and IL-1beta target IL-1alpha for secretion as opposed to surface expression. Proc Natl Acad Sci U.S.A. (2011) 108:18055–60. doi: 10.1073/pnas.1109176108 PMC3207698 22006336

[28] Almog T Kandel-Kfir M Shaish A Dissen M Shlomai G Voronov E. Knockdown of interleukin-1α does not attenuate LPS-induced production of interleukin-1β in mouse macrophages. Cytokine (2015) 73:138–43. doi: 10.1016/j.cyto.2015.01.029 25748836

[29] Copenhaver AM Casson CN Nguyen HT Duda MM Shin S. IL-1R signaling enables bystander cells to overcome bacterial blockade of host protein synthesis. Proc Natl Acad Sci U.S.A. (2015) 112:7557–62. doi: 10.1073/pnas.1501289112 PMC4475993 26034289

[30] Horai R Asano M Sudo K Kanuka H Suzuki M Nishihara M. Production of mice deficient in genes for interleukin (IL)-1alpha, IL-1beta, IL-1alpha/beta, and IL-1 receptor antagonist shows that IL-1beta is crucial in turpentine-induced fever development and glucocorticoid secretion. J Exp Med (1998) 187:1463–75. doi: 10.1084/jem.187.9.1463 PMC2212263 9565638

[31] Gross O Yazdi AS Thomas CJ Masin M Heinz LX Guarda G. Inflammasome activators induce interleukin-1alpha secretion *via* distinct pathways with differential requirement for the protease function of caspase-1. Immunity (2012) 36:388–400. doi: 10.1016/j.immuni.2012.01.018 22444631

[32] Dagvadorj J Mikulska-Ruminska K Tumurkhuu G Ratsimandresy RA Carriere J Andres AM. Recruitment of pro-IL-1alpha to mitochondrial cardiolipin, *via* shared LC3 binding domain, inhibits mitophagy and drives maximal NLRP3 activation. Proc Natl Acad Sci U.S.A. (2021) 118:e2015632118. doi: 10.1073/pnas.2015632118 PMC7817159 33361152

[33] Gurung P Fan G Lukens JR Vogel P Tonks NK Kanneganti TD. Tyrosine kinase SYK licenses MyD88 adaptor protein to instigate IL-1alpha-mediated inflammatory disease. Immunity (2017) 46:635–48. doi: 10.1016/j.immuni.2017.03.014 PMC5501252 28410990

[34] Karki R Lee E Place D Samir P Mavuluri J Sharma BR. IRF8 regulates transcription of Naips for NLRC4 inflammasome activation. Cell (2018) 173:920–933.e913. doi: 10.1016/j.cell.2018.02.055 29576451 PMC5935577

[35] Eigenbrod T Park JH Harder J Iwakura Y Nunez G. Cutting edge: critical role for mesothelial cells in necrosis-induced inflammation through the recognition of IL-1 alpha released from dying cells. J Immunol (2008) 181:8194–8. doi: 10.4049/jimmunol.181.12.8194 PMC2762646 19050234

[36] Sakurai T He G Matsuzawa A Yu GY Maeda S Hardiman G. Hepatocyte necrosis induced by oxidative stress and IL-1 alpha release mediate carcinogen-induced compensatory proliferation and liver tumorigenesis. Cancer Cell (2008) 14:156–65. doi: 10.1016/j.ccr.2008.06.016 PMC2707922 18691550

[37] Di Paolo NC Miao EA Iwakura Y Murali-Krishna K Aderem A Flavell RA. Virus binding to a plasma membrane receptor triggers interleukin-1 alpha-mediated proinflammatory macrophage response *in vivo* . Immunity (2009) 31:110–21. doi: 10.1016/j.immuni.2009.04.015 PMC2759279 19576795

[38] Kono H Karmarkar D Iwakura Y Rock KL. Identification of the cellular sensor that stimulates the inflammatory response to sterile cell death. J Immunol (2010) 184:4470–8. doi: 10.4049/jimmunol.0902485 PMC3094104 20220089

[39] Thornton P Mccoll BW Greenhalgh A Denes A Allan SM Rothwell NJ. Platelet interleukin-1alpha drives cerebrovascular inflammation. Blood (2010) 115:3632–9. doi: 10.1182/blood-2009-11-252643 20200351

[40] Lukens JR Vogel P Johnson GR Kelliher MA Iwakura Y Lamkanfi M. RIP1-driven autoinflammation targets IL-1alpha independently of inflammasomes and RIP3. Nature (2013) 498:224–7. doi: 10.1038/nature12174 PMC3683390 23708968

[41] Hsu LC Enzler T Seita J Timmer AM Lee CY Lai TY. IL-1β-driven neutrophilia preserves antibacterial defense in the absence of the kinase IKKβ. Nat Immunol (2011) 12:144–50. doi: 10.1038/ni.1976 PMC3677078 21170027

[42] Cassel SL Janczy JR Bing X Wilson SP Olivier AK Otero JE. Inflammasome-independent IL-1beta mediates autoinflammatory disease in Pstpip2-deficient mice. Proc Natl Acad Sci U.S.A. (2014) 111:1072–7. doi: 10.1073/pnas.1318685111 PMC3903222 24395802

[43] Lukens JR Gross JM Calabrese C Iwakura Y Lamkanfi M Vogel P. Critical role for inflammasome-independent IL-1beta production in osteomyelitis. Proc Natl Acad Sci U.S.A. (2014) 111:1066–71. doi: 10.1073/pnas.1318688111 PMC3903206 24395792

[44] Gurung P Burton A Kanneganti TD. NLRP3 inflammasome plays a redundant role with caspase 8 to promote IL-1beta-mediated osteomyelitis. Proc Natl Acad Sci U.S.A. (2016) 113:4452–7. doi: 10.1073/pnas.1601636113 PMC4843439 27071119

[45] Eislmayr K Bestehorn A Morelli L Borroni M Walle LV Lamkanfi M. Nonredundancy of IL-1α and IL-1β is defined by distinct regulation of tissues orchestrating resistance versus tolerance to infection. Sci Adv (2022) 8:eabj7293. doi: 10.1126/sciadv.abj7293 35235356 PMC8890706

[46] Place DE Kanneganti TD. Cell death-mediated cytokine release and its therapeutic implications. J Exp Med (2019) 216:1474–86. doi: 10.1084/jem.20181892 PMC6605758 31186281

[47] Lukens JR Gurung P Vogel P Johnson GR Carter RA Mcgoldrick DJ. Dietary modulation of the microbiome affects autoinflammatory disease. Nature (2014) 516:246–9. doi: 10.1038/nature13788 PMC4268032 25274309

[48] Malik A Sharma D Zhu Q Karki R Guy CS Vogel P. IL-33 regulates the IgA-microbiota axis to restrain IL-1alpha-dependent colitis and tumorigenesis. J Clin Invest (2016) 126:4469–81. doi: 10.1172/JCI88625 PMC5127671 27775548

[49] Kyriazopoulou E Poulakou G Milionis H Metallidis S Adamis G Tsiakos K. Early treatment of COVID-19 with anakinra guided by soluble urokinase plasminogen receptor plasma levels: a double-blind, randomized controlled phase 3 trial. Nat Med (2021) 27:1752–60. doi: 10.1038/s41591-021-01499-z PMC8516650 34480127

[50] Caricchio R Abbate A Gordeev I Meng J Hsue PY Neogi T. Effect of canakinumab vs placebo on survival without invasive mechanical ventilation in patients hospitalized with severe COVID-19: A randomized clinical trial. JAMA (2021) 326:230–9. doi: 10.1001/jama.2021.9508 PMC8293025 34283183

[51] Shornick LP De Togni P Mariathasan S Goellner J Strauss-Schoenberger J Karr RW. Mice deficient in IL-1beta manifest impaired contact hypersensitivity to trinitrochlorobenzone. J Exp Med (1996) 183:1427–36. doi: 10.1084/jem.183.4.1427 PMC2192516 8666901

[52] Matsuki T Nakae S Sudo K Horai R Iwakura Y. Abnormal T cell activation caused by the imbalance of the IL-1/IL-1R antagonist system is responsible for the development of experimental autoimmune encephalomyelitis. Int Immunol (2006) 18:399–407. doi: 10.1093/intimm/dxh379 16415102

[53] Pelletier S Gingras S Green DR. Mouse genome engineering *via* CRISPR-Cas9 for study of immune function. Immunity (2015) 42:18–27. doi: 10.1016/j.immuni.2015.01.004 25607456 PMC4720985

[54] Bae S Park J Kim JS. Cas-OFFinder: a fast and versatile algorithm that searches for potential off-target sites of Cas9 RNA-guided endonucleases. Bioinformatics (2014) 30:1473–5. doi: 10.1093/bioinformatics/btu048 PMC4016707 24463181

[55] Gurung P Malireddi RK Anand PK Demon D Vande Walle L Liu Z. Toll or interleukin-1 receptor (TIR) domain-containing adaptor inducing interferon-beta (TRIF)-mediated caspase-11 protease production integrates toll-like receptor 4 (TLR4) protein- and Nlrp3 inflammasome-mediated host defense against enteropathogens. J Biol Chem (2012) 287:34474–83. doi: 10.1074/jbc.M112.401406 PMC3464552 22898816

[56] Tweedell RE Malireddi RKS Kanneganti TD. A comprehensive guide to studying inflammasome activation and cell death. Nat Protoc (2020) 15:3284–333. doi: 10.1038/s41596-020-0374-9 PMC7716618 32895525

