# Supplementary figures and images for: Determining distinct roles of IL-1α through generation of an IL-1α knockout mouse with no defect in IL-1β expression
Source: Front Immunol. 2022 Nov 24;13:1068230. doi: 10.3389/fimmu.2022.1068230 (PMC9729281; doi:10.3389/fimmu.2022.1068230)

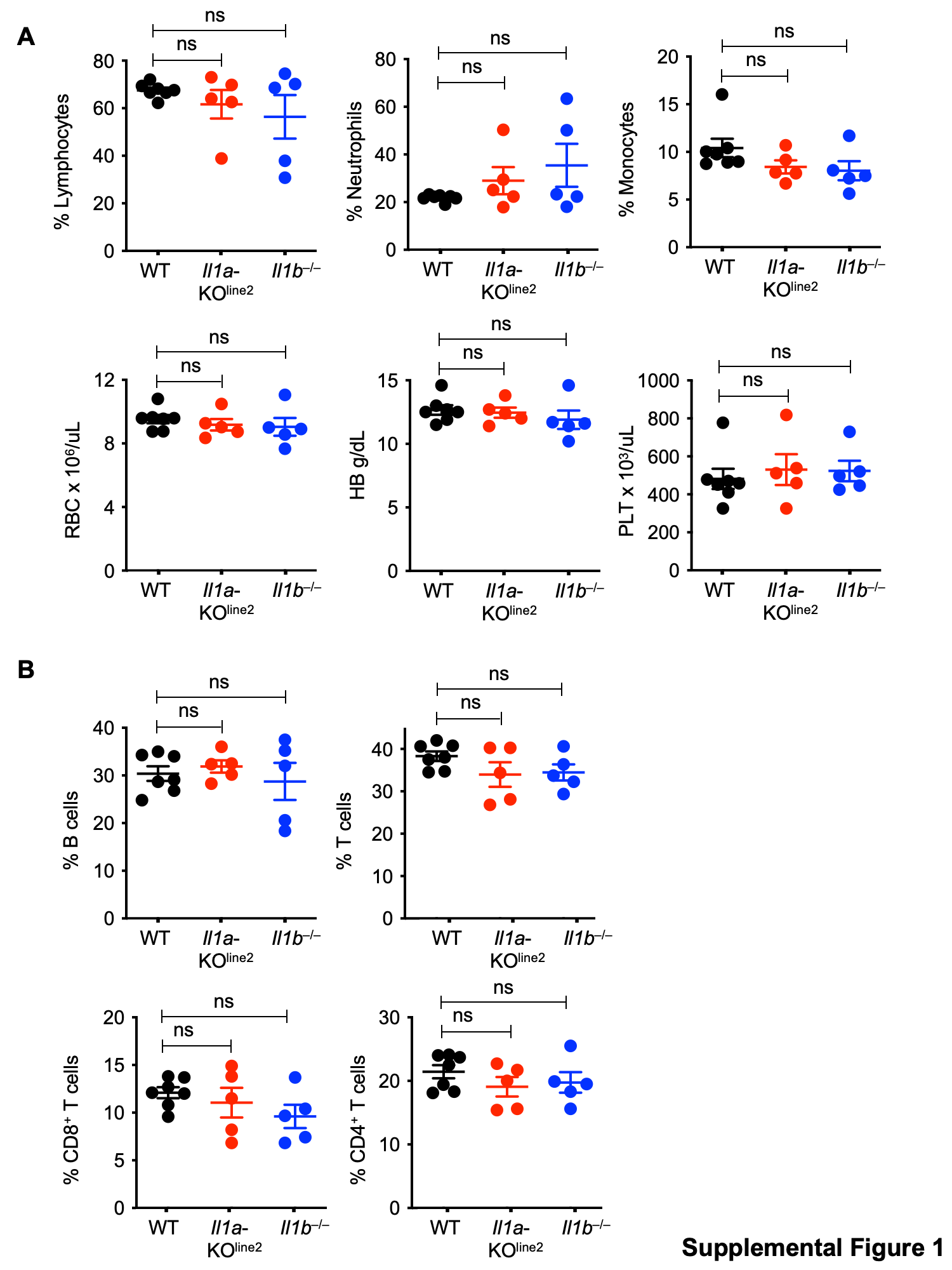

Supplement: Supplementary file 1 [file Image_1.tiff]

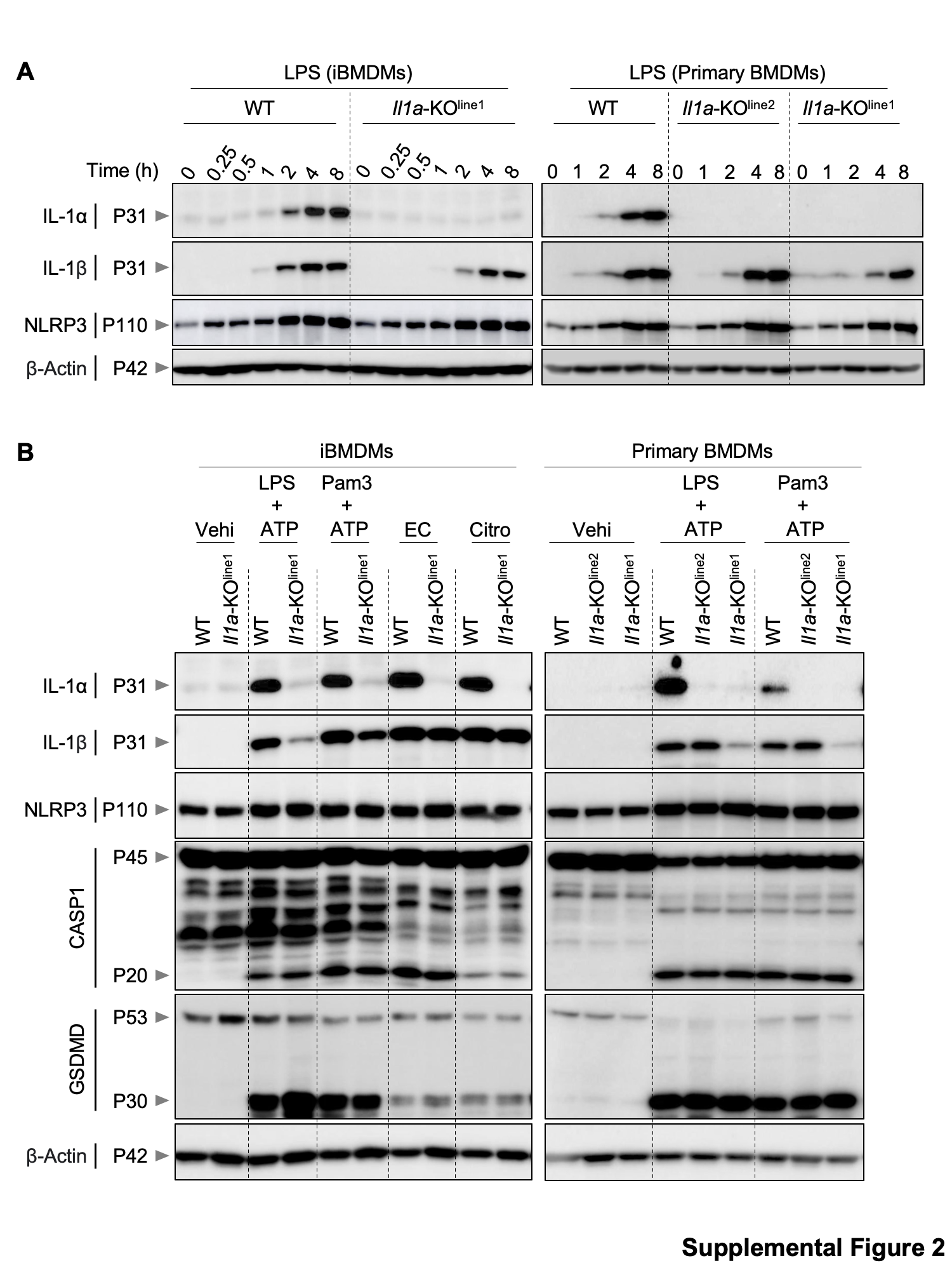

Supplement: Supplementary file 2 [file Image_2.tiff]
